# Shape-controlled synthesis of porous AuPt nanoparticles and their superior electrocatalytic activity for oxygen reduction reaction

**DOI:** 10.1080/14686996.2016.1140307

**Published:** 2016-03-09

**Authors:** Litai Sun, Hongjing Wang, Kamel Eid, Liang Wang

**Affiliations:** ^a^ State Key Laboratory of Electroanalytical Chemistry, Changchun Institute of Applied Chemistry, Chinese Academy of Sciences, Changchun, Jilin130022, PR China; ^b^ University of Chinese Academy of Sciences, Beijing100039, PR China; ^c^ College of Chemical Engineering, Zhejiang University of Technology, Hangzhou310014, PR China

**Keywords:** Keywords, One-step synthesis, metallic nanoparticles, porous structure, catalyst, oxygen reduction reaction

## Abstract

Control of structure and morphology of Pt-based nanomaterials is of great importance for electrochemical energy conversions. In this work, we report an efficient one-step synthesis of bimetallic porous AuPt nanoparticles (PAuPt NPs) in an aqueous solution. The proposed synthesis is performed by a simple stirring treatment of an aqueous reactive mixture including K_2_PtCl_4_, HAuCl_4_, Pluronic F127 and ascorbic acid at a pH value of 1 without organic solvent or high temperature. Due to their porous structure and bimetallic composition, as-made PAuPt NPs exhibit excellent electrocatalytic activity for oxygen reduction reaction.

## Graphical abstract

**Figure:**
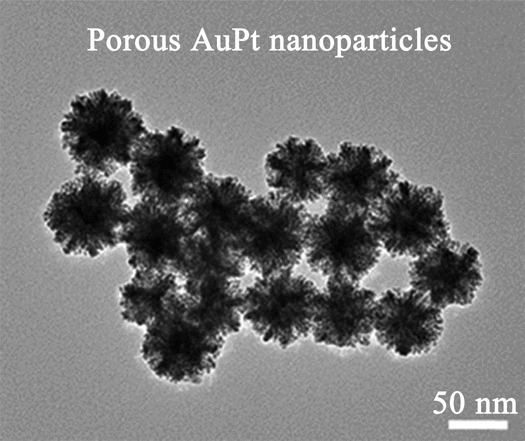

## 1. Introduction

Currently, Pt and Pt-based nanomaterials are the most effective electrocatalysts for oxygen reduction reaction (ORR) and methanol oxidation reaction (MOR) in the proton-exchange membrane (PEM) fuel cell [1–5], which has the properties of high power density and high energy-conversion efficiency. However, the critical issue of the high cost of Pt-based nanomaterials still needs to be addressed. In order to improve utilization efficiency, much effort has been devoted to developing new synthetic methods to obtain Pt-based nanomaterials with composition and structure designed to achieve enhanced performances [6–12].

Among various Pt-based catalysts, bimetallic Pt-based nanomaterials have attracted intensive interesting due to their superior electrocatalytic activity [13–18]. To date, various bimetallic PtM materials with different structures and compositions have been demonstrated. For instance, PtPd nanoicosahedrons [13], hollow PtPd nanoparticles [14], rhombic dodecahedral PtCu nanoframes [15], Au@Porous Pt yolk-shell nanoparticles [16], PtPd nanodendrites [17] and AuPt alloyed flowerlike**-**assembly nanochains [18] have been obtained by different synthetic routes. Generally, control of shapes, composition and surface structures of the nanoparticles can help improve materials’ catalytic properties [19–21].

Porous Pt-based materials are widely used as active electrocatalysts because their porous structures can provide sufficient active sites [22–25]. Their advantages, i.e. high surface area and low cost, make porous Pt-based materials highly desirable, and a lot of effort has been spent preparing them. For example, nanoporous PtFe alloy nanowires are prepared by electrospinning coupled with chemical dealloying [26], PtPd porous nanorods are obtained by a two-step bromide-induced galvanic replacement at high temperature [27], and mesoporous platinum nanospheres are synthesized by using a templated route [28]. These approaches require complicated synthetic procedures, so they are very difficult to scale up. The development of a one-step and efficient method to obtain porous Pt-based nanomaterials remains a great challenge.

Herein, we propose a one-step method for the efficient synthesis of bimetallic porous AuPt nanoparticles (PAuPt NPs). The materials made in this way exhibit superior electrocatalytic activity for ORR compared with commercial Pt/C. The proposed synthetic approach is promising to afford a general strategy for synthesis of interesting bimetallic nanocatalysts by simply adjusting precursor species.

## 2. Experimental details

### 2.1. Chemicals

Commercial carbon-supported Pt catalyst (20 wt%, Pt/C) was purchased from Alfa Aesar[AQ3]; K_2_PtCl_4_, HAuCl_4_, ascorbic acid (AA) and HCl were obtained from Sinopharm Chemical Reagent Co. (Shanghai, China), Ltd; Nafion (5 wt%) and Pluronic F127 ((PEO)_100_(PPO)_65_(PEO)_100_, Mw = 12600) were ordered from Sigma (Missouri, America). The reagents were of analytical grade and were used without further purification. All aqueous solutions were prepared with ultrapure water (>18 MΩ) from a Milli-Q Plus system (Millipore, Massachusetts, America).

### 2.2. Preparation of the porous AuPt NPs

Pluronic F127 (0.02 g) was ultrasonically dissolved in 1.5 ml of K_2_PtCl_4_ (20 mM) and 0.5 ml of HAuCl_4_ (20 mM) aqueous solution, and then drops of 1:1 HCl aqueous solution were added to adjust the pH value to about 1. After adding 2.0 ml of 0.1 M AA as a reducing agent, the mixture was stirred in a water bath for 3 h at 35°C. The final product was collected and washed with water for five times and then dried at 50°C for 24 h for further characterization.

### 2.3. Characterization

Scanning electron microscopy (SEM, FEI/Philips X-L30 field-emission gun setup, from Fei Ltd., Oregon, America) and transmission electron microscopy (TEM, Hitachi H-8100 from Hitachi Ltd. in Tokyo, Japan operated at 100 kV, and JEM 2100F, Jeol Ltd., Tokyo, Japan operated at 200 kV were used to investigate the morphology and structure of the products. X-ray diffraction (XRD) pattern was recorded by using a D8ADVANCE diffractometer (Bruker AXS, Karlsruhe, Germany) with Cu_Ka_ (l = 1.5406 Å) radiation. X-ray photoelectron spectroscopy (XPS) analysis was carried out by using an ESCALAB MK II spectrometer (VG Scientific, Sussex, UK) with Al_Ka_ X-ray radiation for excitation.

### 2.4. Electrochemical investigations

Cyclic voltammetry (CV) was recorded by using a CHI 842C electrochemical workstation (Chenhua Co., Shanghai, China) equipped with a three-electrode system. A rotation disk electrode (RDE, 3 mm diameter) was used as the working electrode, a Pt wire as the counter electrode, and an Ag/AgCl (saturated KCl) electrode as the reference electrode. Prior to the surface coating, the RDE electrode was carefully polished with aqueous alumina suspensions on felt polishing pads.

#### 2.4.1. Preparation of working electrode

The RDE was coated with 3 μl of the catalyst and 3 μl 0.05 wt% Nafion, respectively, to result in a loading of 17 ug cm^−2^_Pt_ for both PAuPt NPs and Pt/C.

#### 2.4.2. Electrochemically active surface area (ECSA)

CV measurements were performed in a N_2_-saturated 0.1 M HClO_4_ solution at a sweep rate of 50 mV s^−1^. ECSA was calculated by using the formula [29,30]:

$$ECSA=\frac{Q_{H}}{m\times q_{H}}$$

where *Q*_H_ is the integrated charge of the hydrogen desorption region after double-layer correction, and *q*_H_ is the charge required for monolayer adsorption of hydrogen on a Pt surface taken as 210 μC cm^−2^.

#### 2.4.3. Oxygen reduction reaction

Before the electrochemical test, the working electrode was first cycled for 50 cycles (–0.2 to 1.0 V at 500 mV s^−1^) in an N_2_-saturated 0.1 M HClO_4_ solution to generate a clean electrode surface. ORR measurements were conducted by using a RDE-3A rotation system (ALS Co., Tokyo, Japan) with a rotation disk electrode in an O_2_-saturated 0.1 M HClO_4_ solution with a rotation speed of 1600 rpm at a scan rate of 10 mV s^−1^. The current densities were normalized in reference to the geometric area of the working electrode, specific and mass activities were normalized in reference to the ECSAs and the loading amounts of Pt, respectively. The Koutecky-Levich equation for the ORR at a RDE is as follows [31]:

$$\frac{1}{i}=\frac{1}{i_{\text{d}}}+\frac{1}{i_{\text{k}}}$$

Here *i* is the measured current, *i*_d_ and *i*_k_ are the diffusion-limiting current and kinetic current, respectively. *i*_k_ was calculated from the following equation:

$$i_{\text{k}}=\frac{i\times i_{\text{d}}}{i_{\text{d}}-i}$$

The durability tests were performed at room temperature in an O_2_-saturated 0.1 M HClO_4_ solutions by applying cyclic potential sweeps between 0.33 and 0.83 V at a sweep rate of 10 mV s ^−1^ for a given number of cycles.

## 3. Results and discussion

**Figure 1.**
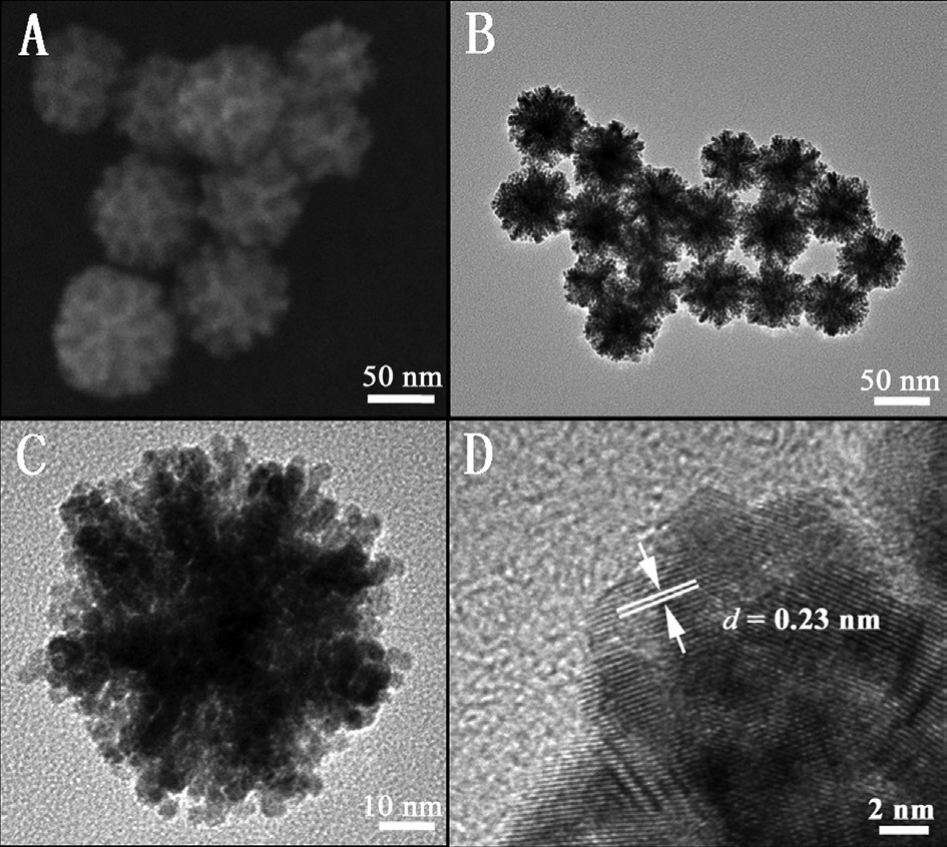
(A) SEM image and (B, C, D) TEM images of the PAuPt NPs.

SEM and TEM were used to investigate the morphology and structure of the PAuPt nanoparticles. Figure 1(A) clearly shows that the nanoparticles with an average diameter of 60 nm had porous structures. Figure 1(B) and (C) reveals that the nanoparticles were assembled by small nanoparticles with a size ranged from 2 to 3 nm. The dendritic entity showed concave exteriors, which resulted in the porous structure inside each nanoparticle. In low-angle XRD pattern for PAuPt NPs, a broad peak was observed **(**Figure S1), further revealing their porous structure. The characteristic diffraction peak at around 0.4° implied that the pore size was around 20 nm, which agreed with the SEM results [8]. Figure 1(D) shows a TEM image recorded at the edge of one particle, revealing that the lattice fringe with a *d* spacing of 0.23 nm corresponded to the (111) plane of the face-centered-cube (*fcc*) crystal structure of Pt. In the selected-area electron diffraction (SAED) pattern (Figure S2), the concentric rings from inside to outside could be assigned to (111), (200), (220) and (311) of *fcc* crystal diffractions of metals, which demonstrated the polycrystalline nature of the PAuPt nanoparticles.

**Figure 2.**
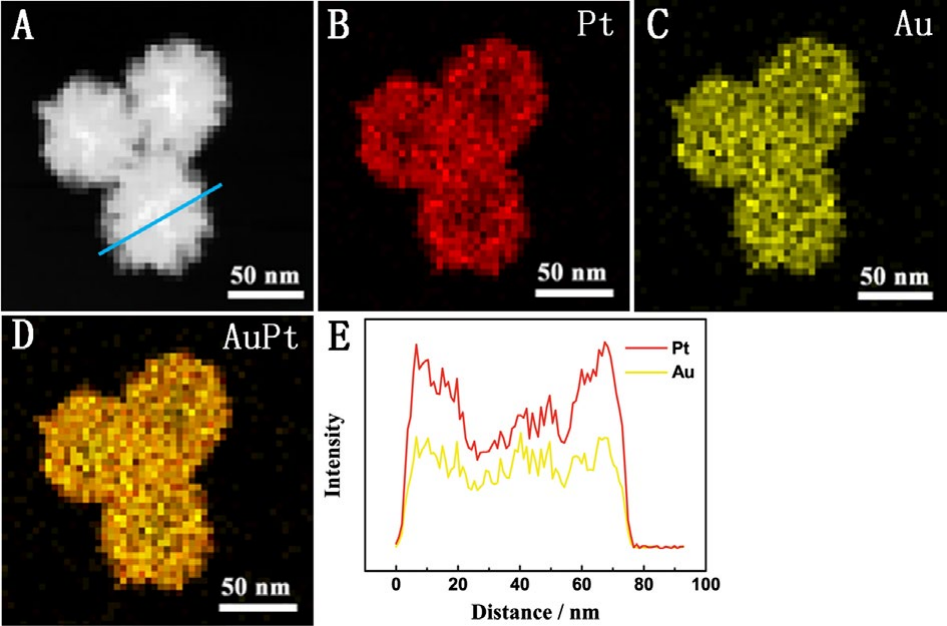
(A, B, C, D) HAADF-STEM-EDS maps of the PAuPt NPs. (E) Cross-sectional compositional line profiles.

**Figure 3.**
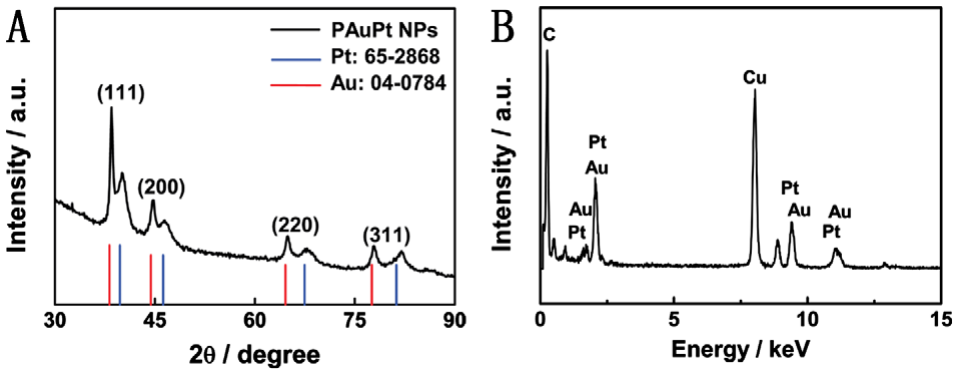
(A) XRD pattern and (B) EDX spectrum of the PAuPt NPs.

High-angle annular dark-field scanning TEM (HAADF-STEM) elemental mappings and cross sectional compositional line profiles clearly revealed that the two elements (i.e. Au and Pt) were uniformly distributed over the particles (Figure 2), implying that the particles were alloy structures. XRD pattern of the PAuPt NPs are displayed in Figure 3(A). The diffraction peaks of PAuPt NPs were indexed to the (111), (200), (220) and (311) planes of Au and Pt, exhibiting an *fcc* structure of Pt and Au, which was coincident with the SAED pattern [17]. The energy-dispersive X-ray (EDX) spectrum further confirmed that the existence of Au and Pt in the nanoparticles (Figure 3(B)). The atom ratio of Au and Pt in the PAuPt NPs was about 1:3, which was determined by inductively coupled plasma optical emission spectrometry (ICP-OES) measurements, suggesting a complete reduction of the metallic precursors.

Based on these investigations, PAuPt NPs have been successfully prepared through a simple stirring treatment of an aqueous reactive mixture including K_2_PtCl_4_, HAuCl_4_, Pluronic F127 and ascorbic acid at a pH value of 1. The formation of porous structures could be explained by the role of the surfactant and the proper reduction rate. In this synthetic system, F127 micelle severed as a template to direct the porous metallic formation [3,8]. The selected precursor ratio of Au and Pt (1:3) was favorable for the high quality synthesis. Adjusting the precursor ratio of Au and Pt resulted in different nanostructures. For example, when the precursor ratio of Au and Pt was 1:1, dendritic structures were obtained, and when the precursor ratio of Au and Pt was 3:1, irregular structures were synthesized (Figure S3). Under the selected pH value of 1, the reduction ability of AA was decreased, resulting in an appropriate rate for metallic atom addition, which was important for the pore formation [3,8].

Porous Pt-based materials have been previously prepared by various methods. Most of the reported methods used complex and non-scalable multi-step procedures [26–28]. The present synthesis showed obvious advantage in one-step procedure without the need of organic solvent and high temperature.

**Figure 4.**
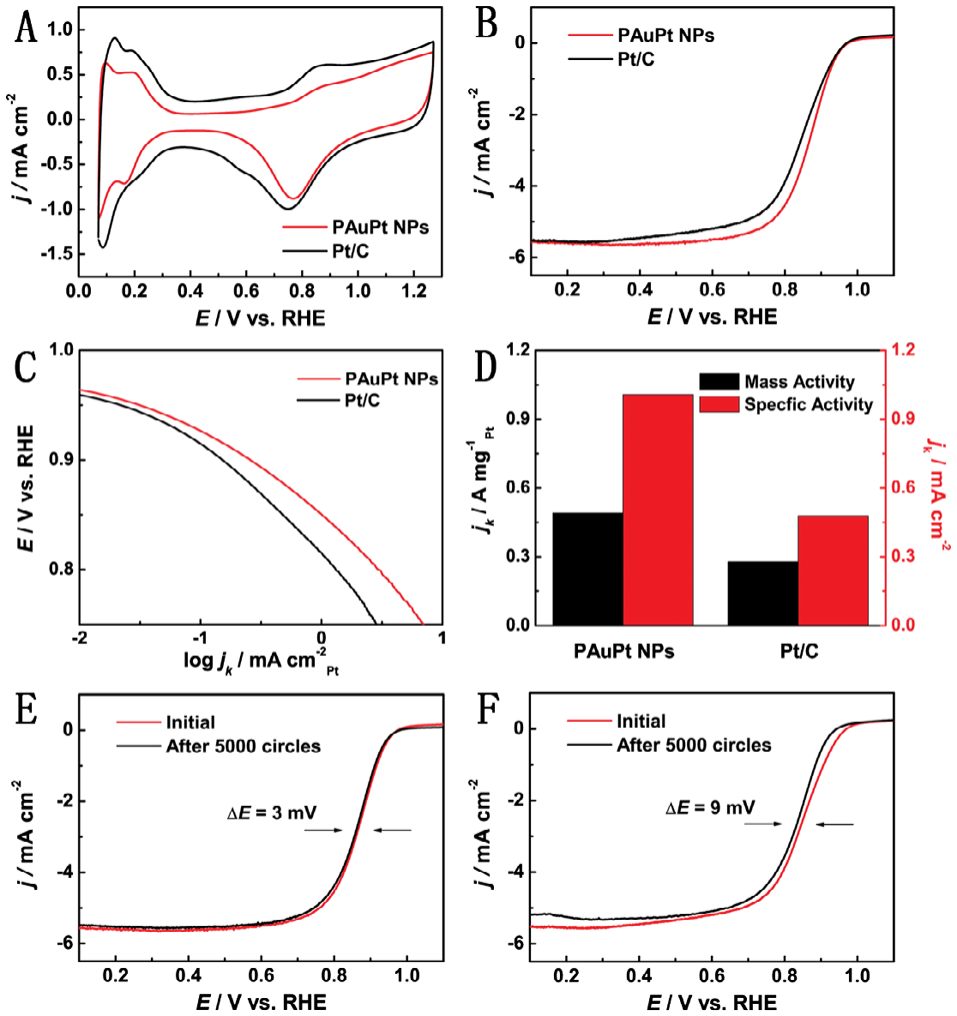
(A) CV . (B) ORR polarization curves of the PAuPt NPs and Pt/C catalyst measured in a N_2_- and an O_2_-saturated 0.1 M HClO_4_ solution, respectively. (C) Corresponding Tafel plots. (D) Comparisons of the specific and mass activities at 0.85 V. (E, F) The ORR polarization curves of (E) PAuPt NPs and (F) Pt/C catalyst, before and after the durability test.

The PAuPt NPs were tested as a catalyst for ORR. ECSA was an important factor for the activity evaluation of the catalysts. Figure 4(A) shows the CVs of PAuPt NPs and Pt/C in a N_2_-saturated 0.1 M HClO_4_ solution which was used to measure the ECSA of the catalysts. The ECSA of PAuPt NPs was estimated to be 49 m^2^ g^−1^_Pt_ which was higher than those of the porous platinum nanochains (25.43 m^2^ g^–1^) [25] and mesoporous Pt nanospheres (16.34 m^2^ g^−1^_Pt_) [28]. It was noted that the ECSA of PAuPt NPs was less than that of the Pt/C (58 m^2^ g^−1^_Pt_), which might be attributed to their larger size. Figure 4(B) displays ORR polarization curves of the catalysts, which consisted of a mixed kinetic-diffusion control region (from 1.0 to 0.7 V) and a diffusion-limiting region (below 0.7 V). The onset potential of PAuPt NPs (0.976 V) was more positive compared that of Pt/C (0.972 V). The half-wave potential PAuPt NPs (0.865 V) was also more positive than that of Pt/C catalyst (0.842 V).

As shown in Figure 4(C), the kinetic current densities of the PAuPt NPs were larger than that of Pt/C catalyst during mixed kinetic/diffusion region. The mass activities and specific activities of the two catalysts were further compared (Figure 4(D)). The mass activity of PAuPt NPs (0.491 A mg^−1^) was higher than that of Pt/C (0.278 A mg^−1^) and the specific activity of PAuPt NPs (1.008 mA cm^−2^) was also higher than that of Pt/C (0.479 mA cm^−2^). These investigations demonstrated that the PAuPt NPs exhibited a better electrocatalytic activity for ORR in comparison with Pt/C. Durability of the catalysts was further tested. After 5000 cycles, the ORR polarization curves of the PAuPt NPs and Pt/C showed a degradation of 3 and 9 mV in the half-wave potential, respectively (Figure 4(E) and (F)), revealing the better durability of the PAuPt NPs compared with that of the Pt/C. The enhanced ORR activity of the PAuPt NPs might be ascribed to their porous structure and bimetallic Au and Pt compositions [3,8].

## 4. Conclusion

In summary, we have successfully synthesized porous AuPt NPs using an efficient one-step approach. The materials made in this way showed excellent performance for ORR application compared with Pt/C catalyst. Due to their porous structure and bimetallic composition, the porous AuPt NPs might also be suitable for other applications beyond fuel cells, such as analysis and biosensors.

## Notes on Contributors

***Litai Sun*** is PhD Student in state key laboratory of electroanalytical chemistry, Changchun Institute of Applied Chemistry, Chinese Academy of Sciences. Her major research interest is to design novel Pt based nanomaterials for electrochemical applications.

***Hongjing Wang*** is Assistant Professor in College of Chemical Engineering, Zhejiang University of Technology, China. Her research interest is to design nanomaterials for electrochemical applications.

***Kamel Eid*** is PhD Student in state key Laboratory of electroanalytical chemistry, Changchun Institute of Applied Chemistry, Chinese Academy of Sciences. His research interest is to synthesis of nanomaterials for electrochemical applications.

***Liang Wang*** is Professor in College of Chemical Engineering, Zhejiang University of Technology, China. His research interest is to design nanomaterials for electrochemical applications.

## Disclosure statement

No potential conflict of interest was reported by the authors.

## Funding

This work was supported by the National Natural Science Foundation of China [No. 21273218].

Supplemental data for this article can be accessed through Taylor & Francis or from author.

## References

[CIT0001] Jung N, Chung D, Ryu J (2014). Pt-based nanoarchitecture and catalyst design for fuel cell applications. Nano Today.

[CIT0002] Kakati N, Maiti J, Lee S (2014). Anode catalysts for direct methanol fuel cells in acidic media: do we have any alternative for Pt or Pt-Ru?. Chem Rev.

[CIT0003] Ataee-Esfahani H, Liu J, Hu M (2013). Mesoporous metallic cells: design of uniformly sized hollow mesoporous Pt-Ru particles with tunable shell thicknesses. Small.

[CIT0004] Wang YB, Zhao N, Fang B (2015). Carbon-supported Pt-based alloy electrocatalysts for the oxygen reduction reaction in polymer electrolyte membrane fuel cells: particle size, shape, and composition manipulation and their impact to activity. Chem Rev.

[CIT0005] Wang H, Jeong H, Imura M (2011). Shape- and size-controlled synthesis in hard templates: sophisticated chemical reduction for mesoporous monocrystalline platinum nanoparticles. J Am Chem Soc.

[CIT0006] Sun X, Li D, Ding Y (2014). Core/shell Au/CuPt nanoparticles and their dual electrocatalysis for both reduction and oxidation reactions. J Am Chem Soc.

[CIT0007] Wang L, Yamauchi Y. (2011). Strategic synthesis of trimetallic Au@Pd@Pt core-shell nanoparticles from poly(vinylpyrrolidone)-based aqueous solution toward highly active electrocatalysts. Chem Mater.

[CIT0008] Ataee-Esfahani H, Imura M, Yamauchi Y (2013). All-metal mesoporous nanocolloids: solution-phase synthesis of core-shell Pd@Pt nanoparticles with a designed concave surface. Angew Chem Int Ed.

[CIT0009] Eid K, Malgras V, He P (2015). Synthesis of mesoporous Pt films with tunable pore sizes from aqueous surfactant solutions. RSC Adv.

[CIT0010] Wang H, Wang L, Sato T (2012). Synthesis of mesoporous Pt films with tunable pore sizes from aqueous surfactant solutions. Chem Mater.

[CIT0011] Chen G, Zhao Y, Fu G (2014). Interfacial effects in iron-nickel hydroxide-platinum nanoparticles enhance catalytic oxidation. Science.

[CIT0012] Wang L, Wang H, Nemoto Y (2010). Rapid and efficient synthesis of platinum nanodendrites with high surface area by chemical reduction with formic acid. Chem Mater.

[CIT0013] Yin AX, Min XQ, Zhu W (2012). Multiply twinned Pt-Pd nanoicosahedrons as highly active electrocatalysts for methanol oxidation. Chem Commun.

[CIT0014] Wang L, Yamauchi Y (2013). Metallic nanocages: synthesis of bimetallic Pt-Pd hollow nanoparticles with dendritic shells by selective chemical etching. J Am Chem Soc.

[CIT0015] Ding J, Zhu X, Bu L (2015). Highly open rhombic dodecahedral PtCu nanoframes. Chem Commun.

[CIT0016] Zhang H, Wang H, Eid K (2015). Nanoparticle in nanocage: Au@porous Pt yolk-shell nanoelectrocatalysts. Part Part Syst Charact.

[CIT0017] Wang H, Ishihara S, Ariga K (2012). All-metal layer-by-layer films: bimetallic alternate layers with accessible mesopores for enhanced electrocatalysis. J Am Chem Soc.

[CIT0018] He L, Song P, Wang A (2015). All-metal layer-by-layer films: bimetallic alternate layers with accessible mesopores for enhanced electrocatalysis. J Mater Chem A.

[CIT0019] Escaño M (2015). First-principles calculations of the dissolution and coalescence properties of Pt nanoparticle ORR catalysts: the effect of nanoparticle shape. Nano Res.

[CIT0020] Wu J, Yang H (2013). Platinum-based oxygen reduction electrocatalysts. Acc Chem Res.

[CIT0021] Huang X, Zhao Z, Cao L (2015). High-performance transition metal-doped Pt3Ni octahedra for oxygen reduction reaction. Science.

[CIT0022] Zhang H, Jin M, Xia Y (2012). Enhancing the catalytic and electrocatalytic properties of Pt-basedcatalysts by forming bimetallic nanocrystals with Pd. Chem Soc Rev.

[CIT0023] Jeong HJ, Kim JW, Jang DY (2015). Atomic layer deposition of ruthenium surface-coating on porous platinum catalysts for high-performance direct ethanol solid oxide fuel cells. J Power Sources.

[CIT0024] Lai J, Zhang L, Qi W (2014). Facile synthesis of porous PtM (M=Cu, Ni) nanowires and their application as efficient electrocatalysts for methanol electrooxidation. Chem Cat Chem.

[CIT0025] Dutta S, Ray C, Mondal A (2015). Aromaticity driven interfacial synthetic strategy for porous platinum nanostructure: an efficient electrocatalyst for methanol and formic acid oxidation. Electrochim Acta.

[CIT0026] Shui J, Chen C, Li JCM (2011). Evolution of nanoporous Pt-Fe alloy nanowires by dealloying and their catalytic property for oxygen reduction reaction. Adv Funct Mater.

[CIT0027] Ge S, Liu W, Liu H (2015). Colorimetric detection of the flux of hydrogen peroxide released from living cells based on the high peroxidase-like catalytic performance of porous PtPd nanorods. Biosens Bioelectron.

[CIT0028] Li Y, Bastakoti BP, Malgras V (2015). Polymeric micelle assembly for the smart synthesis of mesoporous platinum nanospheres with tunable pore sizes. Angew Chem Int Ed.

[CIT0029] Li X, Liu J, He W (2010). Influence of the composition of core-shell Au-Pt nanoparticle electrocatalysts for the oxygen reduction reaction. J Colloid Interface Sci.

[CIT0030] Wanjala BN, Luo J, Loukrakpam R (2010). Nanoscale alloying, phase-segregation, and core-shell evolution of gold-platinum nanoparticles and their electrocatalytic effect on oxygen reduction reaction. Chem Mater.

[CIT0031] Lim B, Jiang M, Camargo P (2009). Pd-Pt bimetallic nanodendrites with high activity for oxygen reduction. Science.

